# The crossing of two unwound transmembrane regions that is the hallmark of the NhaA structural fold is critical for antiporter activity

**DOI:** 10.1038/s41598-024-56425-3

**Published:** 2024-03-11

**Authors:** Abraham Rimon, Hadar Amartely, Etana Padan

**Affiliations:** 1 Department of Biological Chemistry, Alexander Silberman Institute of Life Sciences, Jerusalem, Israel; 2 Wolfson Center for Applied Structural Biology, Jerusalem, Israel; 3 The Hebrew University of Jerusalem, Edmond J. Safra Campus, Givat Ram, 91904 Jerusalem, Israel

**Keywords:** Sodium, Proton antiporter, Membrane transport activity, *Escherichia coli*, NhaA, NhaA structural fold, Biochemistry, Molecular biology

## Abstract

Cell pH and Na^+^ homeostasis requires Na^+^/H^+^ antiporters. The crystal structure of NhaA, the main *Escherichia coli* Na^+^/H^+^ antiporter, revealed a unique NhaA structural fold shared by prokaryotic and eukaryotic membrane proteins. Out of the 12 NhaA transmembrane segments (TMs), TMs III–V and X–XII are topologically inverted repeats with unwound TMs IV and XI forming the X shape characterizing the NhaA fold. We show that intramolecular cross-linking under oxidizing conditions of a NhaA mutant with two Cys replacements across the crossing (D133C-T340C) inhibits antiporter activity and impairs NhaA-dependent cell growth in high-salts. The affinity purified D133C-T340C protein binds Li^+^ (the Na^+^ surrogate substrate of NhaA) under reducing conditions. The cross-linking traps the antiporter in an outward-facing conformation, blocking the antiport cycle. As many secondary transporters are found to share the NhaA fold, including some involved in human diseases, our data have importance for both basic and clinical research.

## Introduction

Na^+^/H^+^ antiporters are critical to homeostatic processes that regulate intracellular pH and Na^+^ content, and therefore cell volume^1,2^. These antiporters are present in the cytoplasmic and organelles membranes of cells^3^, and several are known to be important in health and in disease^4,5^. Furthermore, Na^+^/H^+^ antiporters are critical for salt resistance in plants, an important trait in view of the spread of arid soils^6^.

NhaA, a member of the large and diverse cation/proton antiporter (CPA) superfamily^7^, is the main sodium/proton antiporter in *E. coli* ^8^. It has homologues in other enterobacteria, including many pathogens^4,9^. Several human CPAs—distant homologues of NhaA—are potential drug targets because their malfunction is associated with essential hypertension^10–12^, diabetes^13^, cancer^4,14–16^, or heart failure^17^.

As a secondary transporter, NhaA harnesses the downhill movement of protons across the plasma membrane to facilitate the uphill antiport of Na^+^ or Li^+^ against their electrochemical gradients^18,19^. NhaA mediates an electrogenic transport with a stoichiometry consisting of two protons entering the cell for each cation that exits per antiporter cycle^18,19^. NhaA action thus renders the bacterial cell resistant to lithium/sodium toxicity even at alkaline pH, when its only driving force is the difference in membrane potential that exists across the membrane^1,2,20^. NhaA has a very high turnover rate (10^3^–10^4^ s^−1^) and is drastically dependent on pH^19,21^, a property it shares with many prokaryotic and eukaryotic antiporters. It is inactive below pH 6.5, and its activity increases with pH, peaking at pH 8.5. The transition from inactive NhaA at acidic pH to active NhaA at higher pH is associated with a conformational change that matches the changes in pH-dependent transport^22,23^.

**Figure 1:**
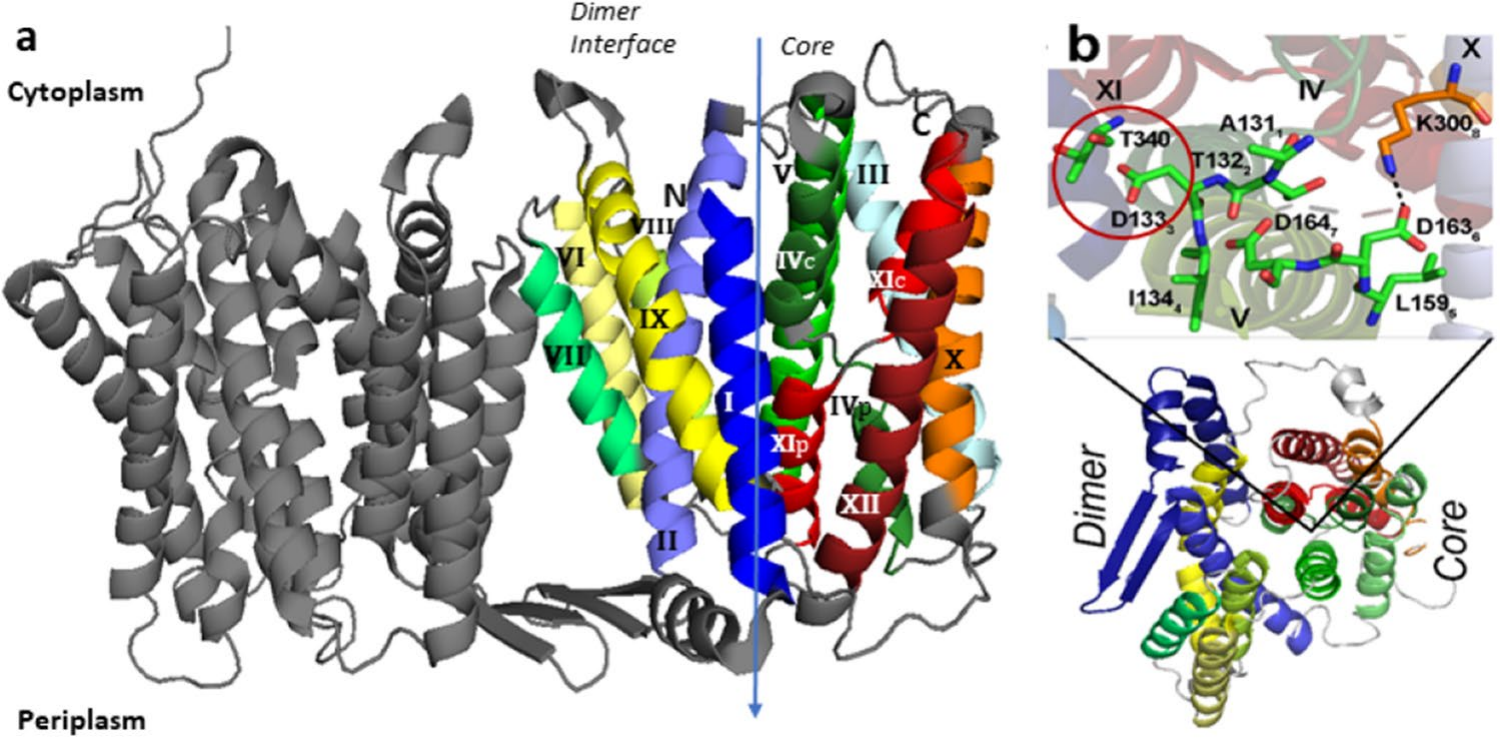
The unique structural fold of NhaA dimer**.** (**a**) Ribbon representation of the crystal structure of the NhaA dimer (pdb:4AU5) viewed parallel to the membrane. Roman numerals denote the 12 TMs. The arrow signifies the border between the two domains, Interface and core. The core displays a view of the NhaA fold signature, in which two antiparallel inverted TMs (IV and XI) are interrupted by their extended chains, which cross each other in the middle of the membrane. (**b**) View of the monomer from the periplasm, with residues D133 and T340 highlighted by a red circle. The subscripts relate to a sequence motif of 8 amino acids, including Asp133 that appears to determine central phenotypic characteristics of the antiporters^52^. The figure was generated using PyMol (The PyMOL Molecular Graphics System, Schrödinger, LLC),

NhaA is a homodimer^24–27^, and following the initial determination of its monomeric crystal structure^28^, the dimer structure was also determined^27^. The monomeric crystal structure^28^ (Fig. 1) revealed a previously unknown structural fold, dubbed the NhaA fold^29^, and opened up the way to structure-based interdisciplinary studies of this class of antiporters.

The functional NhaA monomeric structural fold^30^ is composed of 12 transmembrane helices (TMs)^28^ packed into two domains (Fig. 1): an interface domain (TMs I, II, VI, VII, VIII, and IX) that connects the two NhaA monomers and a core domain (TMs III, IV, V, X, XI, and XII) constituting the functional unit. Between the two domains are two discontinuous funnels open toward the putative ion-binding site, which is thus alternately accessible from either the cytoplasm or periplasm but not both at once, in what is known as an alternating access mechanism^31^.

The core domain is typified by two topologically inverted repeats (TMs III, IV, V and TMs X, XI, XII), of which two (TMs IV and XI) are unwound to extended chains in the middle and cross each other near the center of the membrane (Fig. 1), leaving each with a cytoplasmic-facing (c) and a periplasmic-facing (p) helix (IVc and IVp and XIc and Xip, respectively). This TM assembly with the inverted TMs crossing is the unique hallmark of the NhaA structural fold^29^. Despite considerable sequence diversity, increasing numbers of secondary transporters and other membrane proteins have been found to share the NhaA structural fold, both in prokaryotes^32–37^ and more recently in eukaryotes^12,38–41^.

Nevertheless, the relation between the NhaA structural fold and NhaA function has remained elusive. This is mainly because the atomic structure of NhaA was obtained in inactive form at low pH (pH 3.5–4)^27,28^ and more recently at pH 6.5, at which NhaA is only 10% active^38^.

Using cysteine-accessibility measurements, we found that in EDTA-treated intact cells, D133C of TMIV at the crossing of the extended chain were sensitive to MTSET a membrane-impermeant thiol (SH) reagent, only at alkaline pH. Given its membrane impermeance, MTSET could reach NhaA only at the periplasmic surface in EDTA-treated cells, indicating that the alkylated residue was water accessible and orientated toward the periplasm^23,42,43^. At acidic pH, the periplasmic orientation of D133C was switched to a cytoplasmic orientation, as observed in the crystal structures^23^, making it inaccessible to MTSET. Hence, Asp133 was suggested to function as a periplasmic gate^43^. Taken together, these data indirectly implied that the crossing of the NhaA fold might change position upon NhaA activity.

To test the functional importance of such a change in the crossing of the NhaA structural fold and the associated effects, we constructed a mutant with two Cys replacements at the crossing, D133C-T340C. According to the crystal structures^27,28^, these residues are only 4 Å apart (Fig. 1b)^27,28^. We presumed that in certain NhaA conformers the double Cys replacements, D133C-T340C, would be close enough to cross-link by forming a S–S bond under oxidizing conditions, stalling any movement at the crossing and affecting the associated processes. If any essential step of the antiport cycle and/or its regulation requires an alteration of the spatial relationship between D133C and T340C, oxidative cross-linking would be expected to prevent this change and inhibit the protein’s Na^+^/H^+^ antiporter activity. In contrast, reducing conditions would reverse the inhibition, breaking the disulfide bond and reviving NhaA activity. We indeed found evidence that the crossing of the NhaA structural fold and the associated processes change conformation during NhaA activity, as inhibiting these changes through oxidative cross-linking between D133C and T340C abrogated NhaA function. These results suggest an explanation for the evolutionary conservation of the NhaA structural fold in many membrane ion transporters and other membrane proteins even when they do not share protein sequence.

## Results

### NhaA double Cys replacement mutant D133C-T340C lacks WT capacity to confer E. coli salt resistance under alkaline conditions

**Figure 2:**
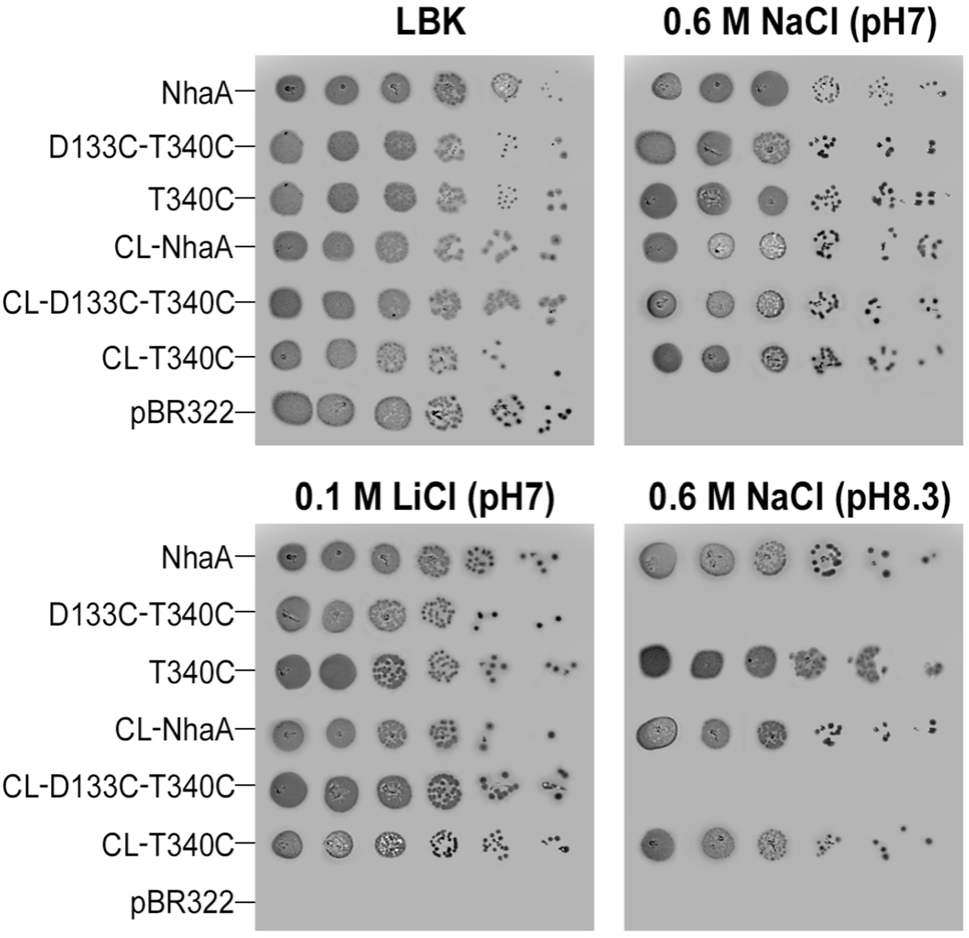
The double NhaA mutant D133C-T340C lacks the capacity to confer salt resistance to cells under alkaline conditions**.** Cells of *E. coli* strain EP432, carrying deletions of both *nhaA* and *nhaB* and transformed with the empty vector pBR322 (negative control), grew only on non-selective medium (LBK, modified LB broth in which NaCl was replaced by KCl) but not on selective media containing 0.1 M Li^+^ (pH 7.0) or 0.6 M Na^+^ (pH 7.0 or pH 8.3). In contrast, similar cells transformed with a plasmid harboring NhaA-WT (positive control) grew on the selective media. Whereas T340C and D133C (not shown) single mutants had salt resistance similar to WT, the double mutant D133C-T340C supported cell growth in the presence of Na^+^ or Li^+^ at neutral pH but not in high Na^+^ at alkaline pH. Note that all plates were grown in open-air, oxidative conditions. All experiments were repeated at least three times, with essentially identical results.

We first tested whether double Cys replacements in proximity to the crossing of the NhaA structural fold would affect the NhaA capacity to confer salt-resistant growth of *E. coli* cells under oxidative conditions. For this purpose, we used two previously constructed single Cys replacements (D133C on TM IV and T340C on TM XI)^44^. As Cys replacements in TM IV are very well studied^42,43^ and result in growth phenotypes on selective media similar to those of the wild type (WT), we Cys-scanned TM XI (Table S1) and showed that the growth phenotype of T340C is also similar to that of the WT. We then constructed paired Cys replacement substitutions, D133C-T340C, in both the Cys-less (CL) NhaA^45^ and WT NhaA backgrounds (we denote the resulting mutants CL-D133C-T340C and D133C-T340C, respectively), and tested the growth phenotypes of the resulting bacteria on selective agar media as compared to the respective single mutants in each background and the WT (Fig. 2).

All cells grew equally on the nonselective medium LBK, and all except the negative control also grew on the moderate selective plates (0.6 M NaCl,pH 7.0). However whereas, the WT and the single mutants also grew in higher selective conditions (0.6 M NaCl, pH 8.3) the double mutant could not. Hence, the double mutant D133C-T340C had lost the capacity to confer salt resistance cells at alkaline pH. Such a loss could be due to reduced protein expression; however, all variants were strongly expressed, at levels similar to those in the WT (Figs. S1 and S4). As the growth was under air (Oxidative conditions), the other likely possibility is that the NhaA-D133C-T340C abolished Na^+^/H^+^ antiporter activity because of cross-linking between the Cys residues.

### Cysteine cross-linking under oxidative conditions inhibits antiport activity of the double mutant D133C-T340C

**Figure 3:**
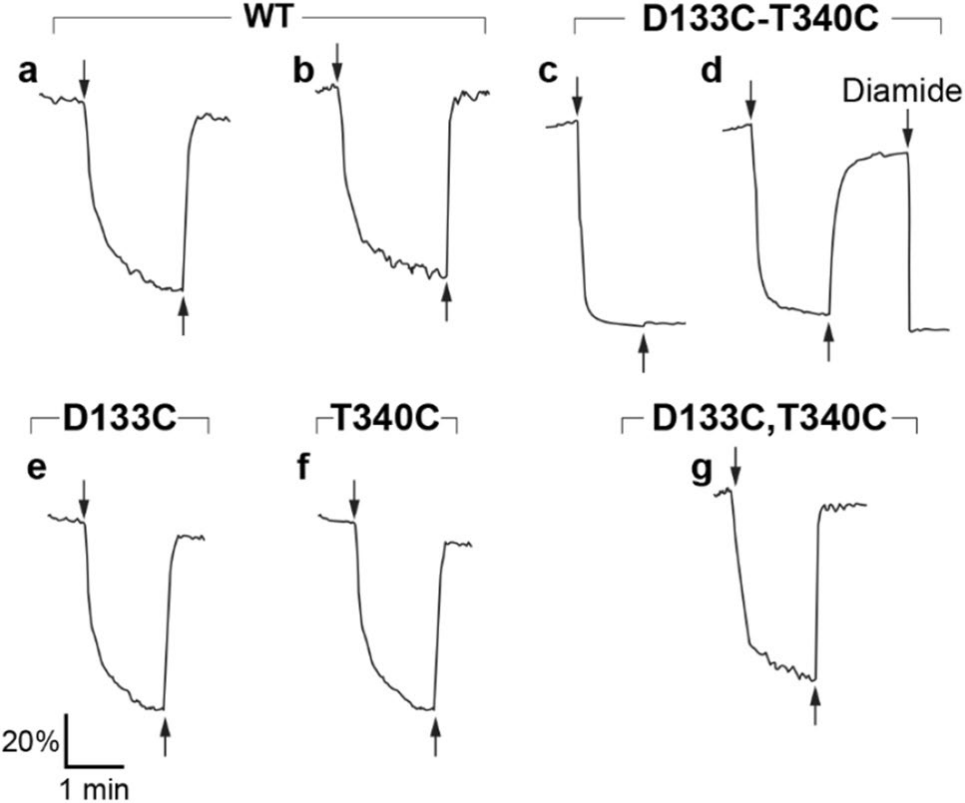
Antiport activity under oxidizing and reducing conditions of inverted membrane vesicles isolated from the EP432 cells expressing variant D133C-T340C as compared to WT and single mutants**.** Everted membrane vesicles were prepared from EP432 cells expressing the indicated NhaA variants grown in LBK, pH 7.5. The Na^+^/H^+^ antiporter activity was estimated in the everted membrane vesicles from the change in ΔpH elicited by the addition of Na^+^, using acridine orange fluorescence as a probe of ΔpH as described in^46^. The reaction mixture contained 150 mM choline chloride, 50 mM BTP (pH 8.5), 5 mM MgCl_2_, 0.5 µM acridine orange, and membrane vesicles (from which 50–100 mg protein was extracted). At the onset of the experiment, Tris D-lactate (2 mM) was added (downward arrow) and the fluorescence quenching was recorded until a steady-state level, representing a steady ΔpH (100% quenching), was reached. NaCl (10 mM) was then added (upward arrow), and the new steady-state level of fluorescence (dequenching) was monitored. (**a**) WT, oxidizing conditions (open air). (**b**) WT, reducing conditions (10 mM DTT or β-Me added 5 min before lactate addition). (**c**) D133C-T340C, oxidizing conditions. (**d**), D133C-T340C, reducing conditions followed by oxidizing conditions (diamide). (**e**), D133C, oxidizing conditions. (**f**) T340C, oxidizing conditions. (**g**) Single mutants D133C and T340C each on different competitive plasmids were transformed together to EP432 cells and the respective membrane vesicles were analyzed for Na^+^/H^+^ antiporter activity as described above. Data from typical experiments are shown and are further described in the text. All experiments were repeated at least three times, with essentially identical results.

To assess the effects of redox/oxidative state on the NhaA-variants' activity, we determined the Na^+^/H^+^ antiporter activity in everted membrane vesicles isolated from EP432 cells expressing D133C-T340C and CL-D133C-T340C, the respective single mutants, or WT at pH 8.5 under both oxidizing and reducing conditions (Fig. 3a–f). As the results of D133C-T340C and CL-D133C-T340C were very similar, we show only the results of D133C-T340C.

We estimated Na^+^/H^+^ antiporter activity from the change in ΔpH elicited by addition of Na^+^ (or Li^+^) using acridine orange fluorescence as a probe of ΔpH, as described in^46^. Under oxidizing conditions (the routine condition, in open air) with the WT (Fig. 3a), we detected the generation of a ΔpH (acidic conditions inside the cell) through oxidation of D-lactate, which resulted in quenching of acridine orange fluorescence. Adding Na^+^ (or Li^+^) resulted in de-quenching, indicating proton influx due to WT NhaA activity. We obtained similar results under reducing conditions (Fig. 3b), created by adding 10 mM DTT or β-Me to the reaction mixture and incubating it for 5 min at room temperature before adding lactate. In marked contrast to the WT, under similar oxidizing conditions, the variant D133C-T340C (Fig. 3c) did not show any antiporter activity. However, under reducing conditions (10 mM DTT or β-Me) antiporter activity was restored to 80% that of the WT (Fig. 3d). Furthermore, later addition of diamide to create oxidizing conditions inhibited the activity (Fig. 3d). The antiporter activity of D133C was similar to the WT with respect to pH sensitivity but the apparent *K*m was increased^43^.

T340C (Fig. 3f) behaved similar to the WT. Panel g. is referred to below.

### Na^+^/H^+^ antiporter activity of D133C-T340C vs. WT as a function of pH

**Figure 4:**
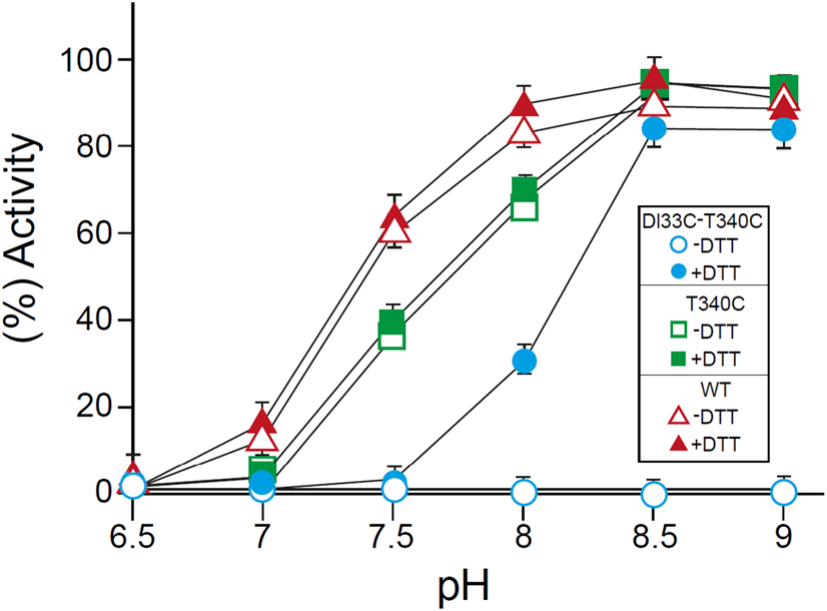
The Na^+^/H^+^ antiporter activity in everted membrane vesicles of D133C-T340C under oxidizing and reducing conditions as a function of pH**.** The experimental conditions (oxidizing and reducing) were as in Fig. 3, but the pH of the reaction mixtures was adjusted to the indicated pH values. The error bars represent standard deviations.

WT NhaA showed the characteristic pH-dependent Na^+^/H^+^ antiporter activity under both oxidizing and reducing conditions: namely, inactivity at pH below 6.5 and activity increasing with pH to a peak at pH 8.5–9.0^19,21^ (Fig. 4). The antiporter activities of the single mutants D133C and T340C were very similar to that of the WT with respect to pH sensitivity but the *K*m of D133C was higher than the WT^43^. In marked contrast to these, the double mutant D133C-T340C had almost no activity (1–2% dequenching) under oxidizing conditions over the pH range 7.5–9 (Fig. 4). However, introducing DTT to the reaction mixture restored the Na^+^/H^+^ antiport activity, which increased gradually from 3% dequenching at pH 7.5 to 80% at pH 8.5 (Fig. 4).

Similar patterns of activity and pH dependence were observed with Li^+^ (data not shown). In summary, the inhibition of the activity of the D133C-T340C variant under oxidizing conditions is caused by cross-linking between its cysteines, which form an S–S bond under oxidizing conditions that is absent under reducing conditions.

### The oxidative inhibitory cross-linking occurs intramolecularly

NhaA’s status as a dimer prompted the question of whether the cross-linking is intramolecular, within NhaA monomers, or extramolecular between the monomers of the dimer and/or between dimers. To address this, we compared the antiporter activity in membrane vesicles isolated from EP432 cells harboring each single mutant (Fig. 3e,f) and cells bearing both single mutants together, each on a different compatible plasmid (Fig. 3g) to that of both mutants in one gene in one plasmid (Fig. 3c,d).

Our results revealed that the single cysteine mutants D133C and T340C were each unaffected by the presence of oxidizing vs. reducing conditions, similar to the WT (Fig. 3a,b). Similarly, when variants D133C and T340C were co-expressed together in the same cells on different plasmids (Fig. 3g), the conditions also had no effect on their antiporter activity. However, when the double mutants were in one gene, the antiporter activity was inhibited under oxidative conditions (Fig. 3c,d). Hence, the cross-linking is intramolecular, because only when D133C and T340C are located in one NhaA gene, in one protein, cross-linking takes place.

### Affinity purification of NhaA D133C-T340C

The longstanding procedure for affinity purification of WT NhaA on Ni^2+^-NTA resin^47^ provides stable, functional NhaA protein that was successfully crystallized and used to determine the protein’s crystal structure at pH 4^28^. Our attempt to apply the same procedure to D133C-T340C mutant NhaA was unsuccessful because the protein precipitated. As this procedure is aerobic, we considered the possibility that the variant protein was becoming cross-linked and therefore precipitating during the purification. Indeed, adding 10 mM β-Me during membrane extraction and later steps (see “Materials and methods” for details) yielded protein that was stable for at least 12 h. The circular dichroism (CD) spectra (Fig. S2a) show that reduced D133C-T340C protein acquires the same secondary structure as the oxidized WT. Addition of β-Me to WT protein leads to slight changes in the helical conformation of the protein. Same conformational changes were observed for the T340C variant at non reducing environment (Fig. S2a). The CD melting curves of the WT at reducing (red) and non-reducing (black) conditions showed that the protein stabilities remained the same. Both T340C (green) and D133C-T340C (blue) mutants were less stable than the WT (Fig. S2b).However, up to 50 °C, all variants were stable.

### Affinity-purified NhaA D133C-T340C binds Li^+^

**Figure 5:**
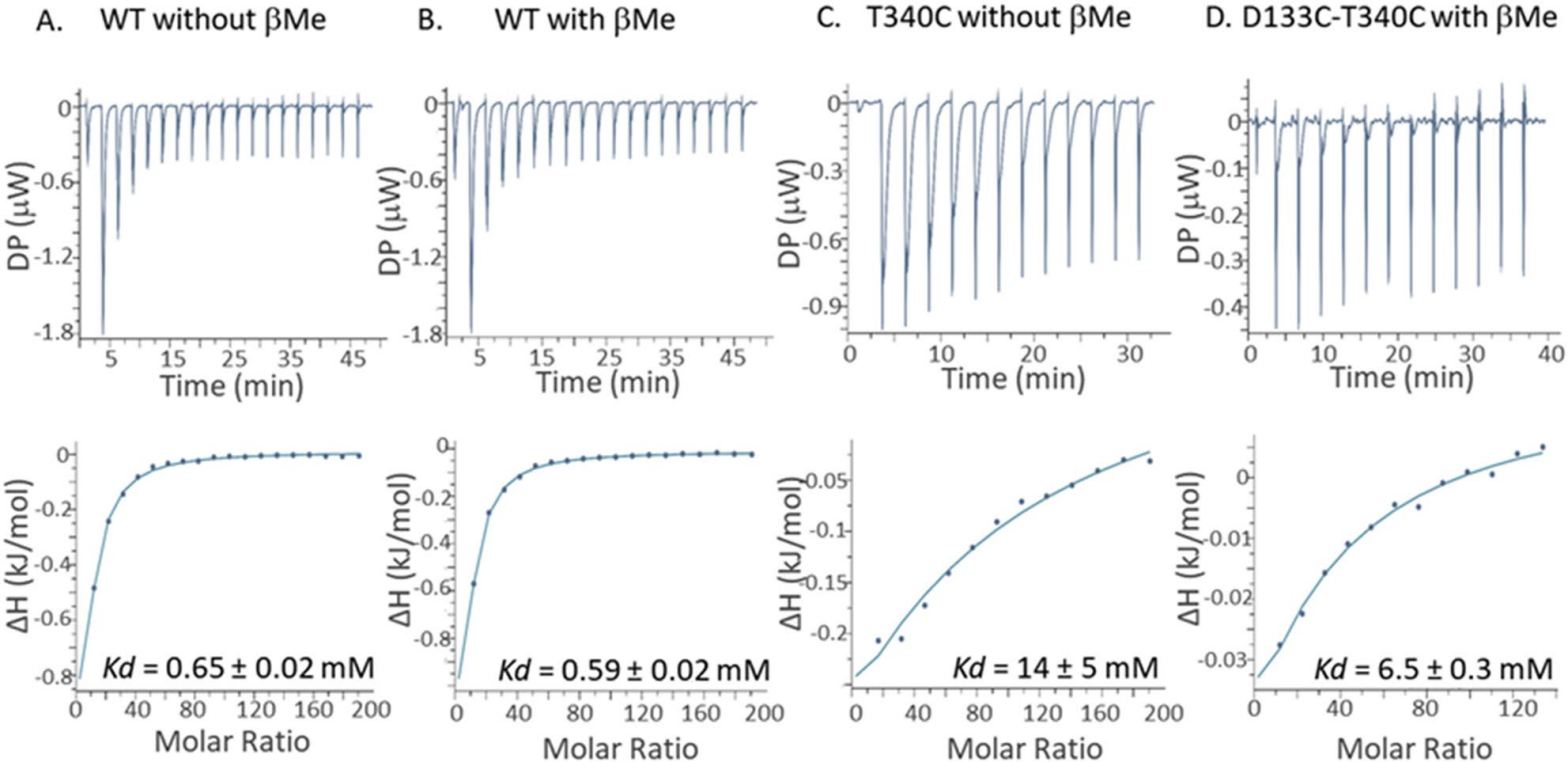
Determination of Li^+^ binding by ITC assays of NhaA variant proteins under oxidizing and reducing conditions**.** Li^+^ binding was tested by ITC to affinity-purified WT at non-reducing (**A**) and reducing (**B**) conditions, T340C at non-reducing conditions (**C**), and D133C-T340C at reducing conditions (**D**) (see “Materials and methods”). Top panels: Heat liberated when 40 mM LiCl solution was injected into the experimental chamber containing ~ 40 µM protein (WT, T340C, or D133C-T340C) at 10 °C in reaction buffer (containing 50 mM BTP, 150 mM choline chloride, 5 mM MgCl_2_, 10% sucrose, 0.05% DDM, with or without 10 mM β-Me, with the pH adjusted to 8.5 using HCl). Bottom panels: Integrated curves representing the total heat exchanged. The blue lines are the best fits to a single-site binding isotherm with N = 1. Experiments were performed 2 or 3 times for WT, T340C, and D133C-T340C, and the binding affinities extracted from the fitting for each replication are reported in Table S2.

Inactivation by the cross-linked mutant D133C-T340C could be due to trapping of the protein in a single conformation that cannot undergo the further changes required to complete the antiport cycle. Another possibility is that the cross-linking degrades the cation-binding site of the variant. We therefore tested Li^+^ binding to the D133C-T340C variant, along with the WT and the two single mutants, using isothermal titration calorimetry (ITC; Fig. 5) under oxidizing and reducing conditions.

The results summarized in Fig. 5 show that WT NhaA bound Li^+^ with *K*_d_ of 0.65 ± 0.02 mM under oxidizing conditions while the T340C mutant bound Li^+^ with *K*_d_ values above 20 fold higher than for the WT. Notably, similar results were obtained previously with mutant D133C^48^. Since D133C-T340C variant was unstable at oxidizing conditions, its Li^+^ affinity was tested at reducing environment and this mutant bound Li^+^ with *K*_d_ value about tenfold higher than for the WT, like the other mutants. To validate that reducing conditions do not affect the binding results, we tested the Li^+^ affinity to the WT protein with addition of β-Me. Same affinity (*K*_d_ = 0.59 ± 0.02 mM) was calculated for the binding of WT NhaA to Li^+^ under reducing conditions.

### Oxidative cross-linking of D133C-T340C antiporter traps it in a conformation different from that of WT NhaA

**Figure 6:**
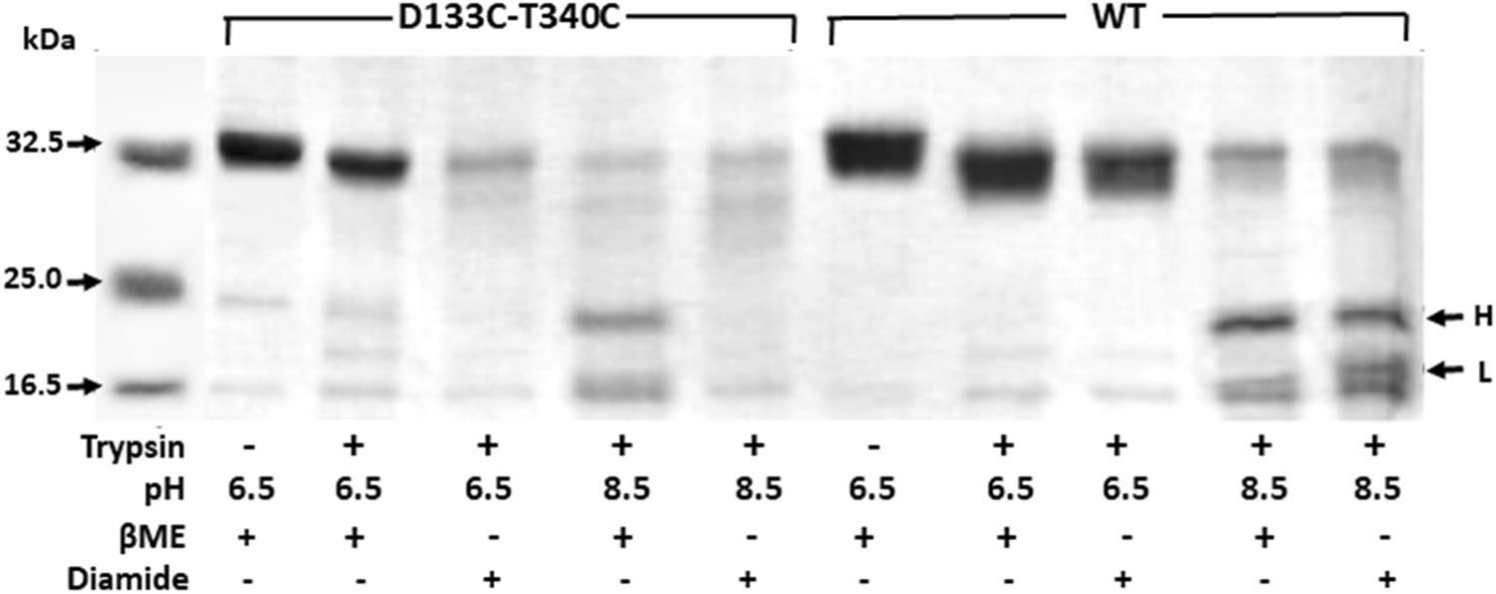
Oxidative cross-linking traps variant D133C-T340C in a conformation different from that of the WT**.** TA16 cells expressing WT or variant D133C-T340C were grown in minimal medium and everted membrane vesicles were isolated as described before. Then, 10 mM β-Me was added in all later steps (protein extraction from the membrane, affinity purification, and storage). To test trypsin digestion, 5 µg of affinity purified protein and 100 ng trypsin (Sigma type III) were used as described in “Materials and methods”. As indicated, certain reaction mixtures contained 10 mM β-Me (reducing agent) or 10 mM diamide (oxidizing agent) and incubated at 37 °C for 1 h. Then, 0.3 µg of the type II trypsin inhibitor was added and the proteins were precipitated in 10% TCA for 30 min at 4 °C. The pellets were collected by centrifugation for 30 min (14,000 rpm, Eppendorf micro-centrifuge), re-suspended in sampling buffer, and resolved by SDS-PAGE. H, heavy peptide; L, light peptide. The leftmost lane contained size standards.

The transition from inactive NhaA (at low pH) to active NhaA (at alkaline pH) is associated with a conformational change demonstrated by trypsin digestion profiles^22^. As for many other proteins, the pattern of trypsin digestion of NhaA provides useful information for identifying conformational changes in the protein^22^. Although NhaA has many trypsin-digestible sites, both the affinity-purified protein and protein in situ in the membrane are exposed to trypsin at acidic pH at only one site, which clips off the NhaA C-terminus. However, another site (Lys249) is subject to trypsin cleavage to form two peptides, heavy (24 kDa) and light (17 kDa), in a pH-dependent fashion that reflects the alkaline pH dependence of the antiporter activity (^22^ and Fig. 6 and Fig. S3). Furthermore, mutants with modified pH dependence were susceptible to trypsin in isolated membrane vesicles only at the pH range in which they were active, with the degree of cleavage reflecting the level of antiport activity. We therefore affinity purified D133C-T340C and WT NhaA under reducing conditions as described above, and tested their trypsin digestion patterns under reducing conditions (with β-Me) and oxidizing conditions (with diamide) (Fig. 6 and Fig. S3). Ref 43 shows the trypsin digestion of D133C. Under both sets of conditions, the WT was slightly cleaved by trypsin at pH 6.5, but was digested into two peptides, heavy (H) and light (L) (24 kDa and 17 kDa, respectively), at pH 8.5^22^. Under reducing conditions (β-Me), the D133C-T340C variant behaved similarly to the WT at both pHs. Under oxidizing conditions (diamide), however, in marked contrast to the WT, D133C-T340C was digested at pH 6.5 (Fig. 6 and Fig. S3) and even more than the WT at pH 8.5. These results imply that cross-linking of variant D133C-T340C changed its conformation as compared to the WT.

### The conformation trapped by D133-T340 NhaA in intact cells at alkaline pH is outward-facing

**Figure 7:**
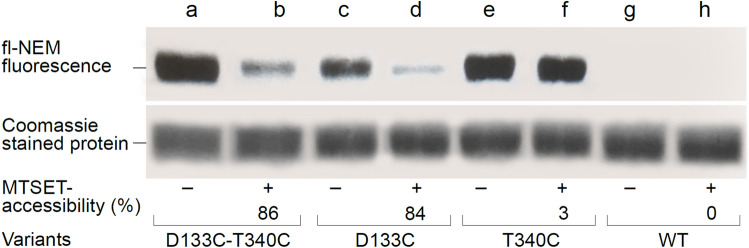
Oxidative cross-linking of D133-T340 mutant traps an outward-facing conformation of NhaA**.** TA16 cells transformed with CL-NhaA harboring variant D133C-T340C (**a**,**b**), D133C (**c**,**d**), T340C (**e**,**f**), or WT cells (**g**,**h**) were either not treated (−) or incubated for 30 min at 23 °C with 10 mM MTSET at pH 8.5 ( +). Then, the NhaA variant from each reaction mixture was purified by Ni^2+^-NTA resin affinity chromatography and labeled on the resin beads with fl-NEM to estimate the percentage of free Cys remaining. The original gel is shown in Fig. S5. Eluted NhaA proteins were resolved by SDS-PAGE, PAGE and Coomassie stained. (The original gel is shown in Fig. S5). The fluorescence level (top panel) and protein concentrations (middle panel) were determined as described in the Materials and Methods. Fluorescence intensity was normalized to the respective protein concentration and expressed as the percent fluorescence intensity of the untreated control (bottom panel). Accessibility to MTSET = 100% − (% fluorescence intensity of the treated sample). The experiment was repeated for three times with similar results.

To test whether this novel conformation is outward- or inward-facing, we used a test for accessibility to the thiol reagent MTSET^49^. MTSET is highly suitable for in situ accessibility studies of single Cys replacements in NhaA^42^, being membrane impermeant, similar in size to hydrated Na^+^, and positively charged. Because of its membrane impermeability, it can reach NhaA in intact *E. coli* cells only if they are treated with EDTA, which perforates the outer membrane^50^. Hence, as described before^42^, MTSET can reach NhaA in EDTA-treated cells from the periplasm or through water-filled pathways in the protein connected to the periplasm. Thus, we treated intact cells of the D133C-T340C mutant, the two single mutants and the WT with EDTA and exposed them to MTSET at pH 8.5 (Fig. 7 and Fig. S5).

The results showed that in both CL-WT and CL-T340C EDTA-treated cells, NhaA is inaccessible to MTSET at alkaline pH. In marked contrast, the CL-D133C-T340C and CL-D133C variants were both highly accessible to MTSET—86% and 84% accessible, respectively (Fig. 7). Hence, at alkaline pH, D133C-T340C stacked the NhaA variant in an outward open conformation very similar to D133C.

In summary, the oxidative cross-linking between D133C and T340C in the D133C-T340C variant constrain NhaA so that it is trapped in one conformation, facing outward, which prevents it from proceeding through the antiport cycle. This points to function-sensitive proximity between Asp133 and Thr340 at the crossing of two unwound helices (TM IV and TM XI) that is the highlight of the NhaA fold.

## Discussion

The crystal structure of inactive *E. coli* NhaA obtained at pH 4^28^ revealed the unique NhaA structural fold (Fig. 1). Out of the 12 transmembrane segments (TMs) comprising NhaA, TMs III–V and X–XII are topologically inverted repeats and TMs IV and XI unwound and cross each other in the mid-membrane to form the X shape characterizing the NhaA fold. Here we show that intramolecular cross-linking under oxidizing conditions of mutant NhaA proteins with two Cys replacement residues near the crossing on TMs IV and XI (D133C-T340C) inhibits antiporter activity (Figs. 3 and 4), impairing NhaA-dependent growth under high-salt conditions (Fig. 2).

The inhibition of the Na^+^/H^+^ antiporter activity of the NhaA-D133C-T340C could be due to trapping of the protein in one stagnant conformation, eliminating its capacity to further change conformation as required for the antiport cycle to proceed. Another possibility is that the cross-linking degrades the cation’s binding site in the D133C-T340C variant. We confirmed that Li^+^ binding by the WT was as previously reported^48^ and was unaffected by the presence of reducing vs. oxidizing conditions (*K*_d_ around 0.6 in both cases) (Fig. 5). We could not determine Li^+^ binding by D133C-T340C mutant under oxidizing conditions because it had precipitated. However, under reducing conditions it bound Li^+^ with a *K*_d_ about tenfold higher than that of WT NhaA (Fig. 5). This high Li^+^ binding *K*_d_ of D133C-T340C was most probably caused by the pair partners D133C and/or T340C. Both single mutants showed tenfold increases in apparent *K*_d_ for Li^+^ (^48^, Fig. 5). In line with this, the apparent *K*_m_ for Na^+^/Li^+^ antiport of D133C (3.6 mM and 1.24 mM) was higher than that of the WT (0.2 mM vs. 0.02 mM), with no significant effect on the pH profile of NhaA activity^43,44,51^.

Taking these results together, it seems most likely that the oxidative cross-linking between D133C and T340C traps NhaA-D133C-T340C in a conformation that cannot proceed in the antiport cycle. As reflected by trypsin digestibility, oxidative cross-linking of the variant D133C-T340C stacked the antiporter in a conformation that is different from that of the WT (Fig. 6). In contrast to the WT, which—except for the C-terminus—is resistant to trypsin at acidic pH under either reducing (β-Me) or oxidizing conditions (diamide), variant D133C-T340C is resistant to trypsin at acidic pH under reducing conditions but fully digested by trypsin at acidic pH in oxidizing conditions (Fig. 6).

To test whether this novel conformation is outward- or inward-facing, we used a test for accessibility of NhaA variants to MTSET in intact cells at alkaline pH. As described before, this SH reagent can reach NhaA only from the periplasm in EDTA-treated cells^42^. The results showed that in both CL-WT and CL-T340C EDTA-treated cells, NhaA is inaccessible to MTSET. In marked contrast, the CL-D133C-T340C and CL-D133C variants were both highly accessible to MTSET—86% and 84% accessible, respectively (Fig. 7 and Fig. S5). Hence, at alkaline pH D133C-T340C stacked the NhaA variant in an outward open conformation very similar to that of D133C. Interestingly, a similar change in accessibility was previously observed with CL-NhaA-D133C at pH 8.5, but not at pH 6.5 (Fig. 2 in^43^). Importantly, the latter accessibility change was reversible upon addition of 10 mM DTT, which removed the MTSET^43^. Similar to the CL-WT, CL-NhaA-T340 was not accessible to MTSET (Fig. 7) because, as observed in the crystal structure, it faces the cytoplasm^28^. Importantly, D133C is active^48^ as opposed to the double mutant which is inactive under oxidative conditions when it cross linked. Hence, we suggest that at alkaline pH the double mutant variant similar to D133C, moves outward. At this outward facing conformation it can be bound by MTSET (Fig. 7) or by cross-linking under oxidative conditions, thereby trapping the antiporter in an outward-facing conformation avoiding continuation of the cycle and inhibiting antiport activity (Figs. 3, 4).

Indeed, Asp133 is one of the most evolutionarily conserved residues of NhaA^23^. Comprehensive evolutionary analysis of 6537 Na^+^/H^+^ antiporters revealed a sequence motif of 8 amino acids, including Asp133 that appears to determine central phenotypic characteristics of the antiporters^52^. Previous results showed that Asp133 has triple roles in NhaA^43^: (i) the negative charge of the Asp133 side chain is critical to the stability of the NhaA structure due to its location between the ends of TM IVc and Xip, compensating for their opposing partial positive dipoles (Fig. 1)^28^. Uncharged replacements for Asp133 abrogate activity and reduce stability of NhaA^43^. Accordingly, the cross-linked D133C-T340C mutant was unstable and precipitated under standard (oxidizing) conditions, but was stable under reducing conditions (see above). We do not know which residue compensates for the partial dipoles of TM IVc and XIp under this condition. However, D133C is more stable than D133A possibly because it is polar and more hydrophilic. (ii) The main chain of Asp133 is part of the cation active site. Alkylated D133C reduces activity but still binds Li^+^ with increased *K*_d_^48^. Because Asp133 is located in the extended chain of TM IV (Fig. 1), its peptide bond is not saturated by hydrogen bonds and it therefore can provide either its carbonyl or the amide of the main chain to coordinate the cation. Molecular dynamics simulations have also shown that Asp133 traps the Na^+^^27^. Notably, a homologue residue to Asp133 in PaNhaP from *Pyrococcus abyssi* binds Tl^+^^35^. (iii) Asp133 is part of an alkaline-pH-dependent gate, which changes the protein’s conformation from an inward-facing conformation to an outward open conformation, and opens the periplasmic funnel at alkaline pH. Thus, at alkaline pH, D133C is inhibited by MTSET, which alkylates D133C from the periplasm^43^, as we show here (Fig. 7). A huge hydrophobic barrier separates the cytoplasmic funnel from the periplasmic funnel^28^, hence, we think that in addition to D133, more residues form the gate to the periplasm. To identify these residues more structural and functional studies are required.

The important role of the D133C side chain under certain conditions has also been shown by simulation^53^. We also show here that adding a Cys replacement T340C, in the other extended chain forming the crossing of the NhaA structural fold (Fig. 1), the double mutant D133C-T340C cross-link under oxidizing condition and inhibit NhaA activity. Hence, inhibition of NhaA activity is caused either by D133C-MTSET or oxidative cross-linked D133C-T340C. Our results clearly show that the latter inhibition is caused by trapping the antiporter in a stagnate outward facing conformation stopping the NhaA cycle. We do not know whether D133C-MTSET inactivates NhaA in a similar fashion because it is also possible that MTSET alkylated D133C is stacked and cannot function as a gate to the periplasm funnel. A huge hydrophobic barrier separates the cytoplasmic funnel from the periplasmic funnel^28^ hence, we think that in addition to D133, more residues form the gate to the periplasm. To identify these residues more structural and functional studies are required.

T340 is also evolutionarily conserved^51^. It is located in a highly conserved patch in TMXI including Leu334, Gly338, Ser342, and Thr340 that is vicinal to TM IV at the crossing. Many mutations in this part of TM XI affect antiporter activity^51^. However, the Cys replacement mutant T340C behaves like the WT^51^ (Fig. 7 and Table S1)*.* Hence, according to our results, oxidative cross-linking between D133C and T340C in the D133C-T340C variant traps the protein in the outward-facing conformation and degrades the conformational change at the crossing a step required for the antiport cycle.

Given that NhaA is a secondary transporter, its overall antiport mechanism follows the canonical alternate accessibility mechanism^31^. The active site is exposed to either the cytoplasmic or periplasmic side of the membrane in an alternating fashion, binding 2 H^+^ on one side of the membrane and exchanging the protons for either Na^+^ or Li^+^ on the other side^18,19^. Hence, to catalyze Na^+^, Li^+^/H^+^ antiport, NhaA must exist in at least two conformations to allow alternating access of the cation-binding site to either side of the membrane. Nevertheless, this overall mechanism involves sequential processes leading to conformational changes, some of which are still under debate related to the mechanism through which cation/proton antiporters alternate between their outward- and inward-facing conformations in the membrane. It is unclear whether the transition of NhaA is driven by a rocking-bundle motion or an elevator motion of the core units with respect to the interface domains^54^. We have used meta-dynamics simulations of two bacterial NhaA proteins, TtNapA from *Thermus thermophilus* and EcNhaA from *E. coli*, which provided support for the rocking-bundle mechanism as the driver of this conformational change^55^.

With respect to the proton donors/acceptors, one suggested pattern is based on a more recently determined crystal structure of NhaA^27,56^, in which Lys300 forms a salt bridge with Asp163. In this model, Na^+^ binds first to Asp164 through competition with the H^+^ of protonated Asp164, and the first H^+^ is expelled. Then, the Na^+^ breaks the salt bridge, releasing the second proton from Lys300, and binds to both Asp163 and Asp164. In a second pattern, which is based on the original crystal structure^28^, Asp163 and Asp164 bind the Na^+^, and each releases an H^+^; here Lys300 is mainly critical for NhaA stability^57^. Our results presented here can accommodate both mechanisms of conformational change or H^+^ donation/acceptance.

In summary, we show here that the crossing of the unwound TM IV and TM XI, the hallmark of the NhaA fold, is required to change its spatial position during activity. Thus, hindering this change through oxidative cross-linking between D133C and T340C at the crossing, in variant D133C-T340C, inhibits NhaA activity by stopping required steps of the antiporter cycle. It is clear that atomic structures of the fully active NhaA (at pH 8.5) would be highly desirable as a means to link all partial steps and resolve the NhaA mechanism.

Our detailed investigation of NhaA adds invaluable information about how best to mutate this protein in future studies. Specifically, at acidic pH, the crossing of TM IV and TM XI, in the middle of the membrane, blocks the cytoplasmic funnel, which is separated from the periplasmic funnel by a long (16-Å) barrier composed of densely packed nonpolar residues in TM II, IVp, and XIp^28^. We presume, based on experimental data^58^, that at physiological (i.e., slightly alkaline) pH, the barrier changes to enable access to the periplasmic funnel. Therefore, it will be necessary to determine the contributions of various amino acids in the hydrophobic barrier between the cytoplasmic and periplasmic funnels at the crossing of the structural NhaA fold, which possibly gate the outward transport pathway together with Asp133.

Although the prokaryotic NhaA is evolutionarily remote from eukaryotic sodium/proton exchangers (NHEs and NHAs), increasing numbers of secondary transporters are being found to share the NhaA structural fold in prokaryotes^32–36^ and recently in eukaryotes^12,37–41^. Therefore, this study should provide insight into the mechanisms by which various naturally occurring mutations in human NhaA homologs lead to defective activity and diseases and could thus facilitate the rational design of drugs.

## Methods

### Bacterial strains and culture conditions

EP432 is a derivative of *E. coli* K-12 with the genotype *mel*BLid, Δ*nha*A1::*kan*, Δ*nha*B1::*cat*, Δ*lac*ZY, *thr*1^20^. TA16 is *nha*A^+^ *nha*B^+^ *lac*I^Q^ and otherwise isogenic to EP432^21^. Cells were grown in either Luria broth (LB) or modified Luria broth (LBK) ^59^. Where indicated, the medium was buffered with 60 mM 1,3-bis-[tris(hydroxymethyl)methylamino] propane (BTP). For plates, 1.5% agar was used. For induction, the cells were grown in minimal medium A^60^ without sodium citrate and with 0.5% (w/v) glycerol, 0.01% (w/v) MgSO_4_·7H_2_O, and 2.5 μg/ml thiamine. Antibiotic was 100 μg/ml ampicillin. Survival of EP432 cells^20^ expressing NhaA variants at high concentrations of Na^+^ or Li^+^ was assessed as previously described^48^.

### Plasmids

pAXH (previously called pYG10) encodes His-tagged WT-NhaA^45^. pAXH-D133C encodes His-tagged NhaA-D133C^42^. pCL-AXH3 is a derivative of pCL-AXH2^44^ and contains a silent BstXI site at position 248 in *nha*A. It encodes Cys-less (CL) His-tagged NhaA. pCL-AXH3-D133C encodes CL-His-tagged NhaA-D133C (denoted herein CL-NhaA-D133C). The pBSTX-T340C plasmid (whose construction has been described previously^61^) encodes T340C without the His-tag. Therefore, its Mlu1–Mun1 fragment was replaced with the equal-sized fragment of pCL-AXH3 or pAXH3 to create pCL-AXH3-T340C or pAXH3-T340C. The double mutant was constructed by replacing the Mlu1–Mun1 fragment of pAXH-D133C with the Mlu1–Mun1 fragment of pBSTX-T340C, providing pAXH-D133C-T340C. The CL variant was obtained similarly using pCL-AXH-D133C, creating pCL-AXH-D133C-T340C.

### Isolation of membrane vesicles and assay of Na^+^/H^+^ antiporter activity

EP432 cells transformed with the respective plasmids were grown in LBK, and everted vesicles were prepared and used to determine Na^+^/H^+^ antiporter activity, as described previously^46^. The assay of antiporter activity was based on the measurement of Na^+^-induced changes in pH as measured by acridine orange, a fluorescent probe of ΔpH. The fluorescence assay was performed with 2.5 ml of reaction mixture containing 50–100 mg of membrane protein, 0.5 µM acridine orange, 150 mM choline chloride, 50 mM bis–tris propane, and 5 mM MgCl_2_, and the pH was titrated with HCl. After energization with D-lactate (2 mM), fluorescence quenching was allowed to achieve a steady state, and then Na^+^ was added. A reversal of the fluorescence level (dequenching) indicated that protons were exiting the vesicles.. As shown previously, the end level of dequenching provides a good estimate of antiporter activity, and the concentration of the ion that gives half-maximal dequenching is a good estimate of the apparent *K*_m_ of the antiporter. The concentration range of the cations tested was 0.01–100 mM at the indicated pH values, and the apparent *K*_m_ values were calculated by linear regression using a Lineweaver–Burk plot.

### Affinity purification of NhaA variants

Overexpression of the NhaA variants, excluding D133C-T340C, was performed as described previously^22,48^ if not indicated otherwise. As the mutant D133C-T340C precipitated under oxidizing conditions, 10 mM β-mercaptoethanol was included in the membrane extraction step and all later steps as follows: 50 mg thawed frozen membrane protein in 5 ml TSC buffer (10 mM Tris, 0.25 M sucrose, and 150 mM choline chloride, pH 7.5) was supplemented with 20% glycerol, 0.1 M MOPS (pH 7), 10 mM β-Me, and 1% β-dodecyl-D-maltoside (DDM) in a total volume of 11.5 ml. The suspension was incubated for 2 h at 4 °C with gentle mixing and then centrifuged for 30 min (278,000* g*) at 4 °C. The supernatant was mixed with 5 ml of pre-washed Ni^2+^-NTA agarose beads (Qiagen) and incubated overnight with gentle agitation at 4 °C. The loaded beads were then washed in binding buffer and washing buffer (Qiagen protocol), but both containing 10 mM β-Me. The protein was eluted in 1.5 ml of elution buffer (10 mM β-Me, 300 mM imidazole, 25 mM citric acid, 100 mM choline chloride, 5 mM MgCl_2_ and 0.015% DDM pH 7.8). The eluted sample was run on desalting column (Hitrap Desalting CYTIVA) with buffer (50 mM BTP, 150 mM choline chloride, 5 mM MgCl_2_, 10% sucrose, 0.015 DDM, pH 8.5, and 10 mM β-Me). The protein (in about 2.5 ml volume) was concentrated by filtration (Amicon Ultra, 30 K) to 40 µM protein in 0.05% DDM. The purified protein was stable for at least 12 h.

### EDTA-treated cells

The EDTA treatment of cells followed the protocol of Leive^50^. Cells expressing the indicated plasmid NhaA variants were washed twice with 120 mM Tris (pH 8) and concentrated 40-fold to 20 OD_600_. After incubation for 1 min at 37 °C, 0.5 mM potassium EDTA was added and incubation was continued for another 2 min. The EDTA-treated cells were washed in 100 mM potassium phosphate, pH 8.5, for treatment with SH reagents.

### Test of accessibility of NhaA Cys replacements in EDTA-treated cells to MTSET, a SH reagent

TA16 cells expressing the respective NhaA variants were grown in minimal medium A to an OD_600_ of 0.7 and induced by addition of 0.5 mM isopropylthiogalactoside (IPTG) for 2 h (to OD_600_ of 1). Then the cells were treated with potassium EDTA, washed, concentrated 10 times in 1 ml KPi buffer consisting of 100 mM potassium phosphate and 5 mM MgSO_4_, pH 8.5, and incubated with 10 mM MTSET for 30 min at 23 °C with mild shaking (450 rpm). The reaction was stopped by dilution with 40 mL KPi buffer, pH 8.5, and then centrifuged (10,000* g* for 5 min at 4 °C). The treated cells were washed twice, re-suspended in 1 mL KPi buffer, and sonicated (three times for 10 s each at 4 °C, using a Vebra Cell Model VCX 750). Unbroken cells were removed by centrifugation (10,000* g*), and the membranes were collected by centrifugation (Beckman TLA 100.4, 265,000*g* for 30 min at 4 °C); the membranes were re-suspended in 0.5 mL TSC buffer [10 mM Tris (pH 7.5), 250 mM sucrose, 150 mM choline chloride], and the protein was extracted and affinity-purified^22^ on Ni^2+^-NTA resin (Qiagen) and left bound to the beads. To determine the amount of free Cys remaining after treatment with SH reagents, the beads were washed twice in binding buffer^45^ at pH 7.4 and once in 500 μL sodium dodecyl sulfate (SDS)-urea buffer [6 M urea, 20 mM Tris (pH 7.5), 2% (w/v) SDS, and 500 mM NaCl], re-suspended in 100 μL SDS-urea buffer containing 0.5 mM fl-NEM (Molecular Probes), further incubated for 30 min at 23 °C, and washed again in SDS-urea buffer. Finally, the proteins were eluted in sample buffer containing 300 mM imidazole (without reducing agent) and separated by SDS–polyacrylamide gel electrophoresis (SDS-PAGE). For evaluation of fluorescence intensity, the gels were photographed under UV light (260 nm) as described previously^62^. To quantify proteins, we stained the gels with Coomassie blue and determined the density of the bands. After normalization of the fluorescence intensity to the amount of protein in each band, protein accessibility to MTSET was determined from the difference in the fluorescence of the reagent-treated versus untreated samples (100% fluorescence = 0% accessibility). The standard deviation was around 5%.

### Detection and quantitation of NhaA and its mutated variants in the membrane

Total membrane protein was determined according to Bradford. The expression level of His-tagged NhaA mutants was determined by resolving the Ni^2+^-NTA-purified proteins by SDS-PAGE, staining the gels with Coomassie blue, and quantifying the band densities with Image Gauge software (Fuji)^45^.

### Trypsin digestion

Affinity-purified protein (5 μg) was re-suspended in a 500 μl reaction mixture containing 100 mM choline chloride, 0.7 mM EDTA, 1 mM CaCl_2_, 0.1% DDM, and 50 mM HEPES titrated to the indicated pH values with Tris. After the addition of 100 ng trypsin (Sigma type III), the suspension was incubated for 1 h at 37 °C, and the reaction was terminated by addition of 300 ng of trypsin inhibitor (Sigma, type II). Then, the protein was precipitated in 10% trichloroacetic acid (TCA) for 30 min at 4 °C and centrifuged (14,000*g*, 30 min). The pellet was re-suspended in sampling buffer and resolved by SDS-PAGE.

### Isothermal titration calorimetry (ITC) measurements

ITC measurements were carried out at 10 °C on an isothermal titration calorimeter (MicroCal PEAQ-ITC). 40 mM Li^+^ was titrated into samples containing 40–50 µM of the WT, T340C, or D133C-T340C variants. All proteins and Li^+^ ions were prepared in a solution containing 50 mM BTP, 150 mM choline chloride, 5 mM MgCl_2_, 10% sucrose, and 0.05% DDM, pH 8.5 with or without 10 mM β-Me. For the titrations, 3 μl aliquots of Li^+^ solution were injected into the protein sample at 4/6 s intervals. A 180-s delay between injections was allowed for equilibration. Titration of Li^+^ ions into the buffer solution was performed as a control and the results were subtracted from the raw data. Analysis were performed using the MicroCal ITC analysis software with fitting to a single-site binding isotherm with 1:1 stoichiometry (N = 1)^48^.

### Circular dichroism (CD) spectroscopy and thermal melting experiments

WT or mutants (6 µM each) were prepared in a solution (pH 7.5) containing 100 mM choline chloride, 5 mM MgCl_2_, 25 mM citric acid, 10% sucrose, and 0.015 DDM, with or without 10 mM β-Me. CD spectra were recorded using a J-1100 spectropolarimeter (Jasco) with a 0.1-cm quartz cuvette for far-UV CD spectroscopy, in a spectral range of 190–260 nm. CD signal values at 220 nm were recorded at increasing temperatures from 20 °C to 90 °C (or 40 °C to 90 °C), with 2 °C intervals between measurements, for WT and mutated variants. To create thermal denaturation curves, CD signal values were transformed to unfolded fraction values using the formula:

1 $$\% unfolded\, = \,\frac{\theta - \theta N\,}{{\theta U\, - \,\theta N}}$$

where θ is the ellipticity at any measured temperature, θ_N_ is the ellipticity at the fully folded state, and θ_U_ is the ellipticity at the fully unfolded state. The data were fit to sigmoidal model using the equation:

2 $$\% unfolded\, = \,1\, - \frac{1}{1\, + \,\exp ((T\, - \,Tm)/t)\,}$$

where *T* is the measured temperature, *T*_*m*_ is the melting point, and *t* is a constant describing the sigmoidal slope.

### Supplementary Information

Supplementary Information.

## Data Availability

All data generated or analysed during this study are included in this published article (and its supplementary information files).

## Abbreviations

CPA: Cation/proton antiporters
TM: Transmembrane segment
BTP: 1,3-Bis[tris(hydroxymethyl)methylamino]propane
LB: Luria broth
Ni–NTA: Ni-nitrilo triacetic acid-agarose
DDM: β-Dodecyl-D-maltoside
MTSES: 2-Sulfonatoethyl methanethiosulfonate
MTSET: [2-(Trimethylammonium) ethyl] methanethiosulfonate bromide
ITC: Isothermal titration calorimetry
β-Me: β-Mercaptoethanol
