# Supplementary material for: The crossing of two unwound transmembrane regions that is the hallmark of the NhaA structural fold is critical for antiporter activity
Source: Sci Rep. 2024 Mar 11;14:5915. doi: 10.1038/s41598-024-56425-3 (PMC10928194; doi:10.1038/s41598-024-56425-3)
Supplement: Supplementary file 1 — Supplementary Information. [file 41598_2024_56425_MOESM1_ESM.docx]

**Supplementary Information**

**Figure S1**


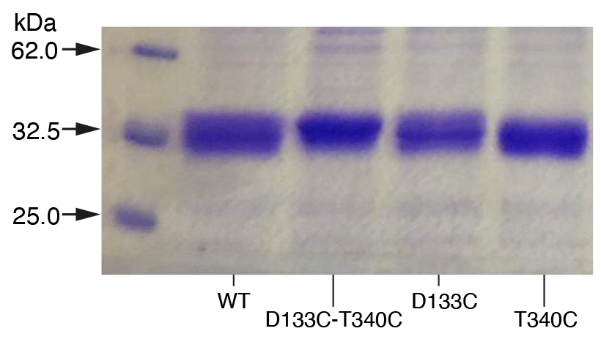


**Figure S1. Protein expression of NhaA variants in isolated membrane vesicles.**

Everted membrane vesicles were isolated from EP432 cells expressing the indicated variants on Ni^-2^-NTA-agarose as described. Equal volumes of the purified proteins were resolved by SDS-PAGE (12.5% SDS) and stained with Coomassie blue.

**Figure S2**


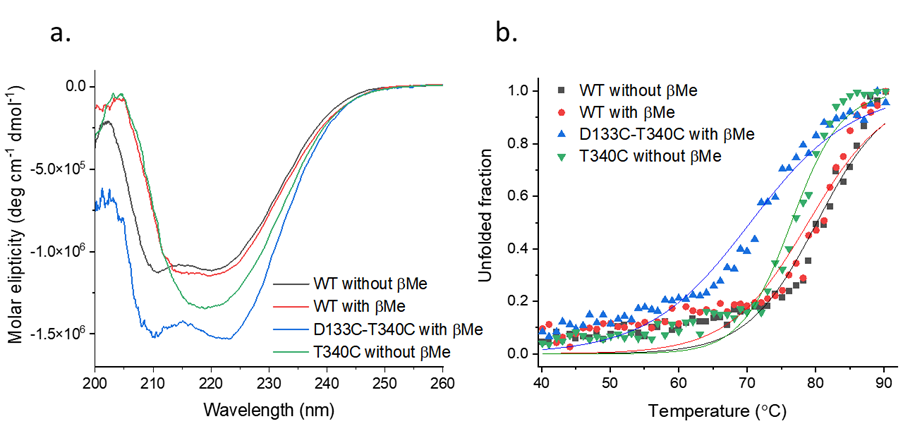


**Figure S2. CD spectra and protein thermal stability of the NhaA variants.** a) CD spectra of WT protein at reducing (red) and non-reducing (black) conditions, T340C mutant at non-reducing conditions (green) and D133C-T340C mutant at reducing conditions (blue). Reduced D133C-T340C mutant acquires the same secondary structure elements as the non-reduced WT protein. Addition of reducing agent to the WT NhaA cause slight change in the secondary structure. Same change was observed for the T340C mutant at non reducing environment. b) CD melting curve as monitored by the change in CD signal at 220 nm. The graph represents the unfolded fraction (see equation 1) of WT NhaA at reducing (red) and non-reducing (black) environment, T340C mutant (green) at non-reducing environment and D133C-T340C mutant (blue) at reducing environment as temperature rises. Melting temperature (*T_m_*) was calculated from the fitted curve (see equation 2); the data are given in Table S2. Stability of WT protein was the same in reducing and non-reducing environments. The stability of the mutant proteins was decreased.

Figure S3 (original Figure 6) **Oxidative cross-linking traps variant D133C-T340C in a conformation different from that of the WT.**

For the experimental procedure see the Legend of Figure 6

Figure S4. (Original Figure S1) **Protein expression of NhaA variants in isolated membrane vesicles.**

For the experimental procedure see Legend S1. The bands shown at 62.0 kDa and above are dimers and polymers respectively.

Figure S5 (Original figure 7)

For the experimental procedure see Legend of Figure 7.


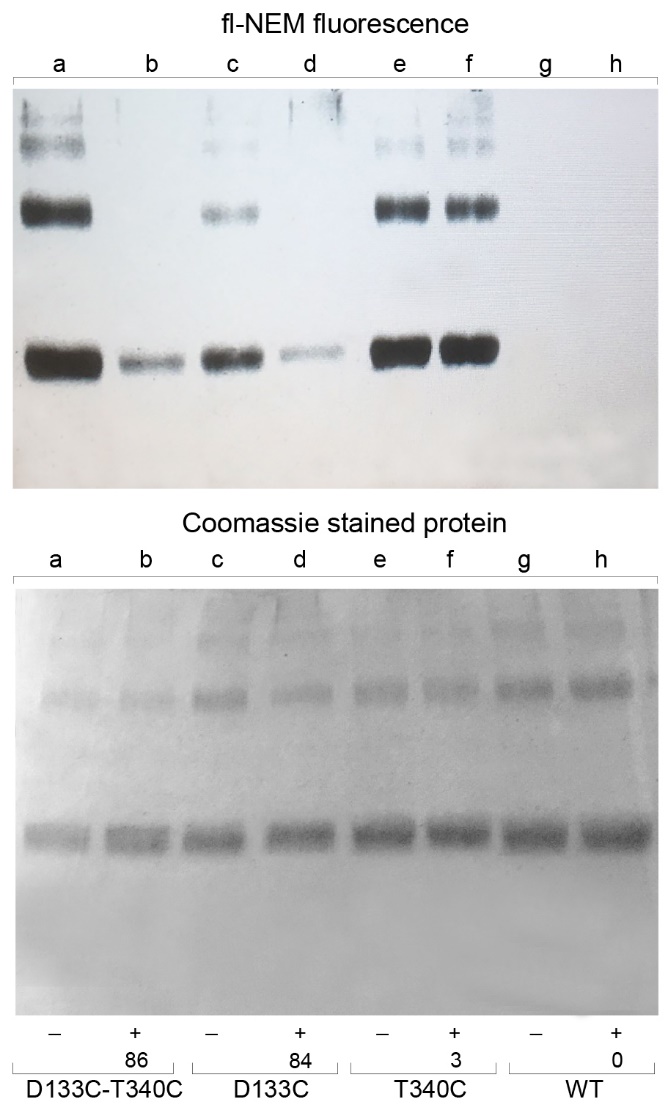


**Table S1. Growth phenotype of Cys replacement of TM XI on selective plates.**

Cells of *E. coli* strain EP432, carrying deletions of both *nha*A and *nha*B, were transformed with a plasmid bearing WT NhaA (positive control) or each of the indicated Cys replacements in TM XI or an empty pBR322 vector (negative control). Growth resistance to Na^+^/Li^+^ was assayed as described in the Materials and Methods section and Figure. 2. ND, not determined. Activity of Na^+^/H^+^ antiporter is expressed as % of the WT.

|  | | 0.6 M NaCl | | | 0.2 M LiCl, pH 7 | Activity (%) | Ref. |
| --- | --- | --- | --- | --- | --- | --- | --- |
|  |  | pH 7 | | pH 8.5 |  |  |  |
| I333C | | + | | + | + | ND |  |
| L334C | | + | | + | + | 100% | {Galili, 2004 #14} |
| S335C | | + | | + | + | ND |  |
| G336C | | + | | _ | + | ND |  |
| I337C | | + | | + | + | ND |  |
| G338C | | _ | | _ | _ | ND |  |
| F339C | | + | | + | + | ND |  |
| T340C | | + | | + | + | 96% | {Galili, 2004 #14} |
| S342C | | + | | + | + | 94% |  |
| F344C | | + | | + | + | ND |  |
| L348C | | + | | + | + | ND |  |
| pBR322 | _ | | _ | | _ | _ |  |

**Table S2. Li^+^ affinity as measured by ITC and melting temperature as calculated from CD melting curves for WT, T340C, and D133C-T340C mutants.**

|  | *K_d_* (mM) | *K_d_* (mM) | *K_d_* (mM) | *T_m_* (°C) |
| --- | --- | --- | --- | --- |
| **WT with β-Me** | 0.65 ± 0.02 | 0.64 ± 0.02 | 0.67 ± 0.01 | 80.2 ± 0.4 |
| **WT without β-Me** | 0.59 ± 0.01 | – | – | 78.9 ± 0.5 |
| **T340C without β-Me** | 17 ± 1 | 13 ± 5 | – | 76.4 ± 0.3 |
| **D133C-T340C with β-Me** | 6.5 ± 0.3 | 4.3 ± 0.5 | – | 70.4 ± 0.4 |
